# Self-Compassion of Nurses Working in Pediatric Hospitals

**DOI:** 10.3390/healthcare14121789

**Published:** 2026-06-21

**Authors:** Dimitra Tsoutsoura, Ioannis Koutelekos, Afroditi Zartaloudi, Areti Stavropoulou, Maria Polikandrioti

**Affiliations:** Department of Nursing, University of West Attica, 11521 Athens, Greece

**Keywords:** self-compassion scale, compassionate care, pediatric nursing, nurses

## Abstract

**Introduction:** Compassion is defined as the emotional response that arises when an individual perceives another’s suffering and is motivated to alleviate it. **Purpose:** To explore levels of self-compassion among nurses working in pediatric hospitals and examine their associations with nurses’ characteristics. **Materials and Methods:** This cross-sectional study included a convenience sample of 208 nurses from a public pediatric hospital. Data were collected through interviews using the Neff Self-Compassion Scale (SCS) which includes the following subscales: Self-Kindness, Common Humanity, Mindfulness, Self-Judgment, Isolation, and Over-Identification. The Greek-validated version of the instrument was used with acceptable internal consistency in the present sample (Cronbach’s alpha = 0.849). Data analysis included descriptive statistics and inferential tests (non-parametric comparisons and multiple linear regression), with statistical significance defined as *p* < 0.05. **Results:** The mean total Self-Compassion score was 83.24 ± 12.6 (range: 26–130). Regarding family-related factors, total Self-Compassion (*p* = 0.029), Common Humanity (*p* = 0.033), and Over-Identification (*p* = 0.041) were associated with the number of children. In relation to age, Self-Kindness (*p* = 0.033), Isolation (*p* = 0.005), and Over-Identification (*p* = 0.005) showed significant associations. Professional factors were also relevant, as Isolation was associated with total years of nursing experience (*p* = 0.032) and choice of nursing as a profession (*p* = 0.004), while Over-Identification was associated with years of experience in pediatric settings (*p* = 0.004) and choice of nursing as a profession (*p* = 0.049). Additionally, marital status was associated with Over-Identification (*p* = 0.045). **Conclusions:** Demographic and professional characteristics appear to influence the expression of Self-compassion. Healthcare organizations should implement targeted training programs to individualize professional development. Future research should explore work-related and personal factors influencing self-compassion to improve care quality and outcomes.

## 1. Introduction

Self-Compassion is defined as the attention and tolerance individuals demonstrate toward themselves during periods of adversity and suffering, as well as the capacity to extend to oneself the same kindness and care that one would offer to others [1,2]. As a psychological construct, Self-compassion reflects the way individuals relate to themselves in situations of failure, inadequacy, or personal distress, adopting an attitude of understanding, kindness, and support rather than self-criticism [3,4].

According to the theory of Self-Compassion which was initially introduced in 2003 by the American psychologist Kristin Neff, the construct encompasses three core dimensions: Self-Kindness versus Self-Judgment, Common Humanity versus Isolation, and Mindfulness versus Over-Identification [1,2]. In more detail, Self-Kindness refers to a caring and understanding attitude toward oneself, as opposed to harsh self-criticism, and involves offering comfort and emotional support during times of difficulty. Common Humanity involves recognizing that imperfection, failure, and mistakes are universal human experiences, thereby situating personal difficulties within a broader shared context. Mindfulness refers to a balanced and non-judgmental awareness of present-moment experiences, in which negative thoughts and emotions are acknowledged without suppression or over-identification. These components interact dynamically, forming a multidimensional system that facilitates adaptive emotion regulation and psychological resilience [4,5].

Having established the conceptual framework, key differences between Self-compassion and related constructs need to be clarified. Self-compassion is distinct from self-pity, which is characterized by a self-focused preoccupation with suffering, perceived helplessness or injustice, and persistent negative affect without adaptive coping [6]. It also differs from compassion in terms of target and conceptualization, as self-compassion is self-directed, whereas compassion is other-oriented. Compassion directed toward friends and family involves providing support within established emotional relationships, while in healthcare and nursing contexts it extends to individuals without prior relational ties, making it a multifaceted process [7,8].

In nursing practice, Self-compassion has been identified as a protective factor against emotional burden and professional burnout [9,10,11]. Higher Self-compassion is associated with greater positive affect, optimism, resilience, and well-being, whereas lower Self-compassion is associated with anxiety, depression, and stress [4,5,8]. Self-compassion may enhance the capacity to provide compassionate care to patients by fostering emotional resilience, reducing burnout, and enabling greater emotional availability in clinical interactions [7,8].

However, in the field of pediatric care, nurses face a wide range of clinical challenges that may hinder the expression of Self-compassion. This is primarily attributable to exposure to patients across diverse developmental stages and frequent involvement in child mortality cases and associated bereavement, both of which impose a substantial psychological burden [12,13]. In addition, pediatric hospital environments are highly stressful for both patients and their families, as child hospitalization is associated with anxiety, uncertainty, and psychological distress [14]. Moreover, traditional nursing norms, policies, media influences, and senior role models often prioritize patient needs, thereby fostering care practices that may undermine Self-compassion [11]. Finally, limited education may hinder the development of Self-compassion, which is regarded as a learnable skill that can be cultivated through education and practice rather than a stable personality trait [15]. Its application remains challenging due to a combination of institutional and individual barriers [11].

Recent research on Self-compassion in nursing has been conducted across diverse populations and clinical contexts, making direct comparisons challenging. For example, Serçe Yüksel Ö et al. (2022) explored oncology nurses’ lived experiences of Self-compassion [16], whereas Salafi et al. (2023) examined Self-compassion among undergraduate nursing students during the COVID-19 pandemic [17]. More recently, Liu et al. (2025) investigated clinical nurses working in tertiary hospitals and demonstrated that Self-compassion was positively associated with work engagement, with moral resilience serving as a mediating factor [9]. Similarly, a large study in New Zealand (n = 1700) involving nurses, physicians, and medical students found that higher self-compassion was associated with lower burnout and better quality of life across all groups [18]. Regarding pediatric nurses, Franco and Christie (2021) evaluated a one-day Self-compassion training intervention aimed at improving resilience among pediatric nurses [19], while Bagewadi et al. (2024) examined the impact of a mindfulness-based intervention on stress among nurses [20]. In contrast, Alanazi et al. (2026) investigated Self-compassion as a moderating factor in the relationship between compassion competence and caring behavior [21]. These findings should be interpreted in the context of the available evidence, particularly considering inconsistencies across studies, methodological limitations in prior research, and the limited body of evidence from pediatric settings.

On this basis, factors associated with Self-compassion among pediatric nurses remain relatively understudied, representing a significant gap in the current literature. This assessment is essential, as it may inform the development of targeted interventions aimed at enhancing pediatric nurses’ psychological well-being and ultimately improving the quality of patient care. To the best of our knowledge, limited evidence exists regarding the levels and determinants of Self-Compassion among pediatric nurses.

The aim of the present study was to explore levels of self-compassion among nurses working in pediatric hospitals and examine their associations with nurses’ characteristics.

## 2. Material and Methods

### 2.1. Study Sample

In this single-center cross-sectional study, 208 pediatric nurses working in a public pediatric hospital in Athens participated between February and March 2025. The hospital provides specialized pediatric care and functions as a referral center for a wide range of acute and complex pediatric conditions, including inpatient, emergency, and intensive care services, characterized by high clinical and emotional demands. Participants were selected using convenience sampling. A total of 228 eligible pediatric nurses were approached to participate in the study. Of these, 208 agreed to participate and completed the questionnaire, corresponding to a participation rate of 91.23%, while 20 nurses declined participation. No a priori sample size estimation or power analysis was conducted prior to data collection.

### 2.2. Inclusion and Exclusion Criteria

The inclusion criteria for participation in the study were (a) age over 18 years, (b) ability to read, write, and understand the Greek language, (c) employment as nursing staff and (d) at least one year of experience in a pediatric hospital/department. Nurses not working in a pediatric setting were excluded from the study.

### 2.3. Data Collection Procedure

Data collection was conducted through face-to-face interviews using a structured questionnaire developed for the purposes of the present study. Completion of the questionnaires required approximately 20–25 min and was mainly carried out when participants were not engaged in patient care activities. Data collection was conducted by a single interviewer to ensure consistency in administration and to minimize interviewer-related bias. Standardized instructions were provided to all participants, and the same procedure was followed for all data collection sessions.

### 2.4. Measurement Instrument

The measurement instrument used was the Self-Compassion Scale (SCS) developed by Neff. The scale also included items assessing the characteristics of pediatric nurses.

#### 2.4.1. Characteristics of Pediatric Nurses

Based on a review of the literature, a structured instrument was developed to assess the characteristics of pediatric nurses. Specifically, the following variables were examined: gender, age, marital status, educational level, number of children, whether nursing was their first career choice, total nursing experience and pediatric experience in years.

#### 2.4.2. Assessment of Self-Compassion

To measure participants’ levels of self-compassion, the Self-Compassion Scale (SCS), developed by Neff (2003), was used [1]. The SCS is the most widely used and theoretically grounded instrument for assessing self-compassion. The full version of the Self-Compassion Scale (SCS) consists of 26 items across six subscales: Self-Kindness, Common Humanity, Mindfulness, Self-Judgment, Isolation, and Over-Identification. The positively worded items are self-kindness, common humanity, mindfulness, while the negatively worded ones are self-judgment, isolation, and over-identification. Responses are rated on a 5-point Likert scale (1 = almost never to 5 = almost always), with higher scores indicating greater self-compassion. The overall score is calculated as the mean of all items after reverse-scoring negatively worded items. The SCS has demonstrated satisfactory psychometric properties in the Greek language [1]. In more detail, internal consistency for the Self-Compassion Scale Greek was acceptable, with Cronbach’s α = 0.87. The subscale reliabilities were as follows: Self-kindness α = 0.70, Self-judgment α = 0.77, Common Humanity α = 0.72, Isolation α = 0.71, Mindfulness α = 0.72, and Over-Identification α = 0.76 [22].

### 2.5. Ethical Considerations

The study was approved by the Scientific Council of the public pediatric hospital, in line with the hospital’s Medical Research Ethics Committee (2nd session, 12 February 2025), and conducted in accordance with the Declaration of Helsinki. Nurses participated after being informed about the purpose of the study, and written informed consent was obtained from all participants. Participation was voluntary and anonymous. Data confidentiality was ensured, and all procedures complied with applicable data protection regulations.

### 2.6. Statistical Analysis

Categorical variables are presented as absolute and relative (%) frequencies, whereas continuous variables are presented as mean values, standard deviations (SD), medians, and interquartile ranges (IQR). The normality of quantitative data was assessed using the Kolmogorov–Smirnov test, as well as graphically through histograms and Q–Q plots. The Kruskal–Wallis test and Mann–Whitney U test were used to examine associations between scale scores and nurses’ characteristics. Subsequently, multiple linear regression analysis was performed to examine the association between participants’ characteristics (independent variables) and Self-Compassion scores (dependent variable).

Before performing multiple linear regression analyses, model assumptions were evaluated. Normality of residuals was assessed using histograms and Q–Q plots of standardized residuals, linearity and homoscedasticity were examined through residual-versus-predicted value plots, and multicollinearity was assessed using tolerance and variance inflation factor (VIF) statistics. Variables demonstrating statistically significant associations (*p* < 0.05) in the univariate analyses were subsequently entered into the multivariable regression models.

Results are presented as beta coefficients and 95% confidence intervals. A *p*-value of less than 0.05 was considered statistically significant. All statistical analyses were conducted using IBM SPSS Statistics, version 28 (IBM Corp., Armonk, NY, USA).

## 3. Results

### 3.1. Sample Description

The study sample consisted of 208 nurses working in a pediatric hospital. The majority were women (91.3%), while only a small proportion were men (8.7%). The largest age group was 41–50 years (27.9%), followed by the 31–35 years group (22.6%), while 18.3% of participants were over 50 years old. Regarding marital status, 58.0% of participants were married, whereas 29.5% were single. In terms of family status, 45.2% had no children, while approximately one in three (30.8%) had one child (Table 1). Concerning professional experience, the majority had 11–20 years of work experience (31.3%), while most participants had been working in a pediatric hospital for 1–5 years (46.6%). Notably, only 39.9% reported that nursing was their first career choice.

### 3.2. Self-Compassion Scale

Table 2 presents the descriptive statistics of the Self-Compassion Scale (SCS) scores for the sample of 208 pediatric hospital nurses. The overall reliability of the scale was high (Cronbach’s α = 0.849), indicating very good internal consistency of the instrument for this population.

The mean total Self-Compassion score was 83.24 (SD = 12.6), within a possible range of 26–130, while the median was 82.0, indicating that 50% of participants scored at or below this value. The interquartile range (IQR) was 76.0–90.0, indicating the distribution of scores within the sample.

For the Self-Kindness subscale, the mean score was 15.98 (SD = 3.6), within a possible range of 5–25, with a median of 16.0 and an interquartile range (IQR) of 14.0–18.5.

For the Self-Judgment subscale, the mean score was 15.49 (SD = 3.6), within a possible range of 5–25, with a median of 16.0 and an interquartile range (IQR) of 13.0–18.0.

For the Common Humanity subscale, the mean score was 12.28 (SD = 2.8), within a possible range of 4–20, with a median of 12.0 and an interquartile range (IQR) of 11.0–14.0.

For the Isolation subscale, the mean score was 13.98 (SD = 3.2), within a possible range of 4–20, with a median of 14.0 and an interquartile range (IQR) of 12.0–16.0.

For the Mindfulness subscale, the mean score was 13.59 (SD = 2.9), within a possible range of 4–20, with a median of 14.0 and an interquartile range (IQR) of 12.0–16.0.

For the Over-Identification subscale, the mean score was 11.93 (SD = 3.4), within a possible range of 4–20, with a median of 12.0 and an interquartile range (IQR) of 10.0–14.0.

The Cronbach’s α coefficients for most subscales were satisfactory, supporting the reliability of the results.

### 3.3. Associations Between Total Self-Compassion and Nurses’ Characteristics

Table 3 shows that the only statistically significant associations between total Self-Compassion (SCS) and the nurses‘ characteristics concern the number of children (*p* = 0.029). All other variables, such as gender, age, marital status, educational level, years of professional experience, choice of profession, and employment status, did not demonstrate statistically significant differences (*p* > 0.05).

Specifically, nurses with more children exhibited higher total Self-Compassion scores. The median total Self-Compassion score was 80 (IQR: 74–88) among participants without children, 83 (IQR: 77–90) among those with one child, and 85 (IQR: 80–97) among nurses with two or more children.

Two statistically significant associations (*p* < 0.05) were observed between Self-Compassion subscales and nurses’ characteristics: Age and Self-Kindness (*p* = 0.033), and Number of Children and Common Humanity (*p* = 0.033). Self-Kindness differed significantly across age groups. Nurses aged > 50 years had the highest median score (18.5, IQR: 15–21), compared with nurses aged ≤ 35 years (16, IQR: 13–18) and those aged 36–40 years (15, IQR: 14–17). Common Humanity was significantly associated with the number of children. Nurses with ≥2 children had a median score of 13 (IQR: 10–15), compared with nurses without children (11, IQR: 9–14). No other statistically significant associations were observed among the remaining subscales or demographic variables (Table 4).

Table 5 presents statistically significant associations between Self-Compassion subscales and pediatric nurses’ characteristics. More specifically:

1. Age and Isolation (*p* = 0.005)
2. Age and Over-Identification (*p* = 0.005)
3. Marital status and Over-Identification (*p* = 0.045)
4. Number of children and Over-Identification (*p* = 0.041)
5. Years of nursing experience and Isolation (*p* = 0.032)
6. Years of experience in pediatric hospitals and Over-Identification (*p* < 0.001)
7. Choice of profession with both Isolation (*p* = 0.004) and Over-Identification (*p* = 0.049).

Associations between Age, Isolation, and Over-Identification:

Nurses aged ≤ 35 years reported higher levels of Isolation with a median score of 15 (IQR: 13–16). The score decreased slightly for ages 36–40 and rose again slightly after 50 years (median 14, IQR: 12–17). Regarding Over-Identification the ≤35 age group had the lowest median of 11 (IQR: 10–13), whereas those aged > 50 recorded the highest median of 14 (IQR: 11–15).

Associations between Marital Status and Over-Identification:

Single nurses had a lower median Over-Identification score (11, IQR: 10–13) compared to married/cohabiting (13, IQR: 11–14) and divorced participants (13, IQR: 12–14).

Associations between Number of Children and Over-Identification:

Nurses with two or more children had a median score of 13 (IQR: 11–15), higher than those without children (12, IQR: 10–13).

Associations between Years of Nursing Total Experience and Isolation:

Nurses with >20 years of total Nursing Experience had the highest median score (15, IQR: 13–17), and those with 11–20 years of experience had the lowest median score (12, IQR: 11–15).

Associations between Years of Pediatric Nursing Experience and Over-Identification:

Nurses with >20 years of Pediatric Nursing Experience had the highest median score (14, IQR: 11–15), compared with other groups whose medians ranged between 11 and 12.

Associations between Choice of Profession, Isolation, and Over-Identification:

Nurses who stated nursing as their 3rd career choice had the highest median in Isolation (16, IQR: 12–17) and a high median in Over-Identification (13, IQR: 11–14) compared with those for whom it was their 1st or 2nd choice (medians 12 and 11, respectively).

The remaining variables did not show statistically significant associations.

### 3.4. Associations Between Self-Compassion and Nurses’ Characteristics Identified Using Multiple Linear Regression Analysis

Multiple linear regression showed that for the total self-compassion score, participants with one child showed higher values compared to those without children (β = 4.01, 95% CI: 0.02 to 7.99, *p* = 0.049), while the increase was even greater for participants with ≥2 children (β = 5.24, 95% CI: 0.93 to 9.54, *p* = 0.017).

In the Self-Kindness subscale, the age group > 50 years presented a significantly higher score compared to the age group < 35 years (β = 1.87, 95% CI: 0.50 to 3.25, *p* = 0.008).

Regarding the Common humanity subscale, individuals with ≥2 children showed marginally higher values compared to those without children (β = 0.95, 95% CI: 0.00 to 1.90, *p* = 0.050).

In the Isolation subscale, the 36–40 year-old age group recorded statistically significantly lower values (β = −1.96, 95% CI: −3.32 to −0.61, *p* = 0.005), while significantly lower scores were also observed among those who had chosen the nursing profession as a third choice compared to those who had it as a first choice (β = −2.29, 95% CI: −3.67 to −0.91, *p* = 0.001).

Finally, in Over-Identification, only choosing the profession as a third choice was significantly associated with lower values compared to those who had it as a first choice (β = −1.90, 95% CI: −3.55 to −0.26, *p* = 0.024) (Table 6).

## 4. Discussion

According to the results of the present study, significant associations were observed between Self-Compassion and participants’ characteristics, thereby addressing the study objectives. From a psychometric perspective, the Common Humanity subscale demonstrated relatively lower internal consistency compared to the other subscales. While previous research has reported adequate reliability for this dimension [1,2,22], the present findings suggest that its measurement properties may vary across different samples and settings.

Regarding Self-compassion scores, nurses demonstrated variations across subscales, with higher scores for Self-Kindness and Self-Judgment, and moderate scores for Common Humanity, Mindfulness, Isolation, and Over-Identification. These findings are presented descriptively and reflect the distribution of Self-compassion dimensions within the study sample. The observed pattern is consistent with the multidimensional conceptualization of Self-compassion proposed by Neff (2003), according to which self-compassion includes both self-compassionate and self-critical components [1,2]. In this context, the coexistence of higher Self-Kindness and Self-Judgment scores suggests that these dimensions may simultaneously be present among nurses in clinical pediatric environments.

Consistent with the present findings, Alanazi et al. [21] reported moderate-to-high scores of Self-Compassion among pediatric nurses in Saudi Arabia. While overall levels were similar, differences were observed in subscale rankings, with Mindfulness highest in Alanazi et al. [21] and Self-Kindness in the present study. Similarly, a study of 961 pediatric nursing students showed Mindfulness the highest-scoring dimension, while Self-Judgment the lowest [23]. Differences across studies may be related to variations in clinical demands, organizational contexts, and coping strategies in pediatric nursing, underscoring the need for a deeper contextual comprehension of Self-Compassion in this population.

Several associations were found between Self-Compassion (and its subscales) and demographic and professional characteristics, including age, number of children, work experience (total and pediatric), marital status, and choice of nursing as a profession. The following section presents an analysis of the factors associated with Self-compassion among pediatric nurses.

Nurses who had two or more children demonstrated higher scores of total Self-compassion. At the subscale level, significant associations were observed only for Common Humanity and Over-Identification, whereas no significant associations were found for the remaining subscales. This finding may be related to differences in life experiences associated with parenting, which may influence emotional awareness and coping in daily and professional contexts. Dual caregiving roles may be associated with adaptive coping and a more balanced response to emotionally demanding situations, as suggested in previous studies [6,24].

Nurses aged over 50 reported higher Self-Kindness and Over-Identification, possibly related to greater professional experience and perceived maturity in coping with occupational stress. In contrast, Upton’s study [7] found Self-compassion to be highest among nurses aged 31–35 years and lowest among those aged 51–55 years, indicating variability in age-related patterns. Continuing with the present age-related findings, nurses aged ≤35 years exhibited a higher Isolation score, suggesting greater endorsement of feelings captured by this subscale. In relevant studies [25,26], age-related differences in Self-compassion dimensions have been reported, with younger nurses showing lower Common Humanity and Mindfulness and higher Over-Identification. However, further research is needed to clarify the relationship between age and Self-compassion.

More experienced participants (>20 years) exhibited higher Over-Identification scores. This pattern is consistent with previous literature reporting higher occupational stress among more experienced nurses and differences in responses to clinical challenges across levels of experience, including the use of skills and experience in managing difficulties [9,27]. Therefore, the relationship between professional experience and Over-Identification appears to be complex and may be influenced by both the increased demands and the enhanced coping resources associated with longer careers.

Divorced nurses demonstrated higher levels of Over-Identification, a subscale reflecting the degree to which respondents endorse items related to becoming emotionally absorbed in negative experiences. This may be associated with differences in social and partner support, which have been reported in the literature to be related to emotional functioning in clinical settings. Social and family support are associated with lower compassion fatigue and higher compassion satisfaction in nursing populations [28].

Isolation was associated with >20 years of total nursing experience, whereas Over-Identification was associated with >20 years of experience in pediatric settings. As previously discussed, this finding may be related to cumulative exposure to occupational stressors over the course of a nursing career. Greater professional responsibilities and repeated exposure to emotionally demanding situations have been described in the literature in relation to feelings of isolation and increased emotional involvement in challenging experiences [29,30].

Participants who reported nursing as a third career choice showed higher Isolation and Over-Identification scores. This may be related to differences in career pathways and prior professional experiences within a profession characterized by substantial workload and emotional demands, which have been reported in relation to career choice and professional adjustment [31].

The present study was grounded on Self-Compassion Theory proposed by Neff, which conceptualizes Self-compassion as a multidimensional construct comprising Self-Kindness, Common Humanity, and Mindfulness. According to this theoretical perspective, Self-compassion has been associated with adaptive emotion regulation, psychological resilience, and effective coping with adversity. The theory further posits that Self-Compassion develops through experiences that shape self-attitudes, emotional responses, and approaches to personal suffering [2,4,5]. From a theoretical perspective, this study applies Self-Compassion Theory to the pediatric nursing context, a clinical setting characterized by substantial emotional demands.

Previous literature suggests that demographic characteristics may influence the expression of Self-Compassion [32,33], but its development is also affected by accumulated life experiences, interpersonal relationships, and broader social and cultural contexts [34]. Within this framework, age, parental, marital and professional status may be related to the expression of Self-Compassion through differences in experiences in challenging life and occupational contexts. The present findings are consistent with this theoretical perspective, as demographic and professional characteristics were associated with Self-Compassion among pediatric nurses.

In more detail, age, family status, and parental experience may be associated with differences in emotional responses within accumulated life experiences, whereas greater clinical exposure has been reported in relation to variations in professional confidence and coping responses. Initial career choice may also be related to differences in perceived preparedness for the emotional demands of nursing and subsequent professional adjustment. Within pediatric clinical practice, individual and professional factors operate in a highly demanding emotional context characterized by repeated exposure to patient suffering, complex family dynamics, and sustained emotional engagement. Such conditions have been reported in relation to higher levels of self-criticism as well as increased vulnerability to compassion fatigue. These effects may be further influenced by limited collegial support and organizational isolation, both of which have been associated with occupational stress [28,35,36,37]. Taken together, these mechanisms suggest that Self-Compassion is shaped by an interplay of personal factors, occupational exposure and accumulated personal experiences within the specific context of pediatric nursing.

Several potential confounding factors should be considered when interpreting findings on Self-Compassion. Among health professionals, Self-Compassion is positively associated with resilience and negatively associated with anxiety and depression, which may influence the observed relationships. Self-esteem may also act as a confounding factor, since higher Self-Compassion is linked to greater perceived competence, reduced fear of failure, and enhanced self-worth [38,39]. Burnout represents another relevant confounder, given its inverse relationship with Self-Compassion and its associations with reduced job satisfaction and poorer sleep quality [39,40]. Finally, prior participation in Self-Compassion training or intervention programs may influence outcomes, as such programs have been shown to increase mindfulness and compassion satisfaction while reducing burnout, anxiety, and stress [19,20].

### 4.1. Limitations of the Study

Several limitations should be considered when interpreting the findings on Self-Compassion among pediatric nurses. The cross-sectional design does not allow for causal inferences between the examined factors and Self-Compassion, while the use of convenience sampling limits the representativeness of the sample and reduces the generalizability of the findings. A priori sample size calculation was not conducted, as the study aimed to include all eligible pediatric nurses available during the data collection period. In addition, the absence of follow-up data precludes the assessment of changes in Self-Compassion over time, which may fluctuate in response to varying work-related conditions. Longitudinal designs would provide a more accurate depiction of this construct in dynamic clinical environments. The absence of a control group is a limitation of the cross-sectional design, which restricts comparative analysis between groups. Furthermore, self-report measures may introduce response bias and may not fully capture actual emotional regulation practices, while the interview-based approach may have introduced interviewer bias. The study was conducted in a single clinical setting in the prefecture of Attica, which may further limit external validity due to potential differences in organizational structures, patient populations, and clinical protocols across settings. A further limitation of this study was the lack of adjustment for potential confounders, including burnout, anxiety, depression, workload, shift work, and organizational support, which may have influenced the observed associations and limited the ability to draw firm conclusions about the independent relationships between the variables under study. Finally, the interpretation of findings may have been influenced by the literature search strategy, which was restricted to studies published in English and Greek and to selected electronic databases.

### 4.2. Clinical Implications and Future Perspectives

Nurses represent the largest segment of the healthcare workforce and maintain continuous patient contact. Self-compassion may function as a key coping resource in clinical settings, enhancing patient-centered care through improved nurse–patient interactions.

At an organizational level, structured interventions aimed at enhancing Self-compassion may support nurses’ psychological well-being and resilience over time. Accordingly, healthcare managers should consider implementing targeted training programs to foster these skills within the workforce. Emerging technologies may further support future interventions by enabling more individualized approaches to professional development.

From a research perspective, strengthening Self-compassion among nurses, particularly in high-demand settings such as pediatrics, should be prioritized to improve healthcare quality, organizational efficiency, and patient outcomes. Future studies should further explore work-related, demographic, and personality factors associated with Self-compassion in pediatric nurses to better understand individual differences in its development and application.

## 5. Conclusions

The findings indicate relatively high self-compassion scores among pediatric nurses. Associations were observed between Self-compassion (and its subscales) and demographic and professional characteristics. Number of children was associated with total Self-compassion, Common Humanity, and Over-Identification. Age was associated with Self-Kindness, Isolation, and Over-Identification. Work experience was associated with Isolation (total nursing experience) and Over-Identification (pediatric experience). Marital status was associated with Over-Identification, while choice of nursing as a profession was associated with Isolation and Over-Identification.

By integrating demographic and professional factors within a single framework, the study provided a more comprehensive understanding of Self-compassion in this clinical setting. The findings highlight the need for the implementation of targeted interventions aimed at enhancing Self-compassion, strengthening psychological support systems, and preventing burnout among pediatric nurses. Such programs may include structured Self-compassion training, stress management strategies, and organizational initiatives designed to promote supportive work environments.

Future longitudinal and multicenter studies are warranted to further examine these associations and establish the directionality of the observed relationships.

## Figures and Tables

**Table 1:** Description of the sample of nurses working in pediatric hospitals (n = 208).

	n (%)
Gender	
Male	18 (8.7%)
Female	190 (91.3%)
Age (years)	
≤30	28 (13.5%)
31–35	47 (22.6%)
36–40	37 (17.8%)
41–50	58 (27.9%)
>50	38 (18.3%)
Marital status	
Married	120 (58.0%)
Single	61 (29.5%)
Divorced	17 (8.2%)
Cohabiting	9 (4.3%)
Number of children	
0	94 (45.2%)
1	64 (30.8%)
2	36 (17.3%)
>2	14 (6.7%)
Total experience (years)	
1–5	31 (14.9%)
6–10	51 (24.5%)
11–20	65 (31.3%)
21–30	37 (17.8%)
>30	24 (11.5%)
Pediatric experience (years)	
1–5	97 (46.6%)
6–10	36 (17.3%)
11–20	44 (21.2%)
>20	31 (14.9%)
Nursing choice (rank)	
1st	83 (39.9%)
2nd	69 (33.2%)
3rd	56 (26.9%)

**Table 2:** Descriptive statistics of the Self-Compassion Scale (SCS) scores (n = 208).

	Cronbach’s α	Mean (SD)	Median (IQR)
Self-Compassion Scale Total (SCS) (Range: 26–130)	0.849	83.24 (12.6)	82.0 (76.0–90.0)
Self-kindness (Range: 5–25)	0.748	15.98 (3.6)	16.0 (14.0–18.5)
Self-judgment (Range: 5–25)	0.688	15.49 (3.6)	16.0 (13.0–18.0)
Common humanity (Range: 4–20)	0.608	12.28 (2.8)	12.0 (11.0–14.0)
Mindfulness (Range: 4–20)	0.722	13.59 (2.9)	14.0 (12.0–16.0)
Isolation (Range: 4–20)	0.640	13.98 (3.2)	14.0 (12.0–16.0)
Over-identification (Range: 4–20)	0.762	11.93 (3.4)	12.0 (10.0–14.0)

**Table 3:** Associations of nurses’ characteristics with total Self-Compassion.

	Self-Compassion Total (SCS)		
	Mean (SD)	Median (IQR)	*p*-Value
Gender			0.344
Male	80.6 (9.5)	80.5 (76–87)	
Female	83.5 (12.9)	83 (76–91)	
Age (years)			0.153
≤35	82.4 (13.9)	81 (74–90)	
36–40	80.5 (12.7)	82 (76–86)	
41–50	83.4 (11.3)	84 (76–89)	
>50	87.4 (11.1)	84 (79–98)	
Marital status			0.226
Married/Cohabiting	84.2 (12.4)	83 (77–90)	
Single	81.1 (13.2)	80 (74–89)	
Divorced	82.8 (11.6)	79 (78–89)	
Number of children			0.029
0	80.7 (12.0)	80 (74–88)	
1	84.8 (12.5)	83 (77–90)	
≥2	86.0 (13.4)	85 (80–97)	
Total experience (years)			0.154
1–5	84.9 (13.6)	84 (76–94)	
6–10	82.6 (13.2)	82 (75–92)	
11–20	78.4 (9.7)	78 (70–84)	
>20	83.8 (10.6)	83.5 (78–89)	
Pediatric experience (years)			0.268
1–5	84.2 (12.6)	82 (75–90)	
6–10	80.5 (13.7)	78 (72–90)	
11–20	83.4 (12.5)	84 (78–89)	
>20	84.9 (11.7)	83 (78–90)	
Nursing choice (rank)			0.166
1st	84.1 (13.5)	82 (76–90)	
2nd	80.2 (12.8)	82 (75–87)	
3rd	81.0 (10.7)	82.5 (73–88.5)	

**Table 4:** Associations of nurses’ characteristics with Self-Compassion subscales (Self-Kindness, Self-Judgment, Common Humanity).

	Self-Kindness		Self-Judgment		Common Humanity	
	Median (IQR)	*p*-Value	Median (IQR)	*p*-Value	Median (IQR)	*p*-Value
Gender		0.132		0.676		0.949
Male	14 (13–16)		16 (13–19)		12 (10–14)	
Female	16 (14–19)		16 (13–18)		12 (10–14)	
Age (years)		0.033		0.917		0.264
≤35	16 (13–18)		16 (13–18)		12 (10–15)	
36–40	15 (14–17)		15 (13–18)		11 (9–13)	
41–50	15 (13–18)		15.5 (13–18)		12 (9–15)	
>50	18.5 (15–21)		16 (12–17)		13 (11–15)	
Marital status		0.581		0.447		0.591
Married/Cohabiting	16 (14–19)		16 (13–18)		12 (10–14)	
Single	16 (14–17)		15 (13–18)		11 (10–14)	
Divorced	15 (14–19)		15 (13–16)		13 (9–15)	
Number of children		0.470		0.313		0.033
0	15 (13–18)		15 (13–18)		11 (9–14)	
1	15.5 (14–18.5)		16 (13–18)		12 (10–14)	
≥2	16.5 (14–20)		16 (14–19)		13 (10–15)	
Total nursing experience		0.648		0.188		0.091
1–5	16 (14–19)		16 (14–18)		12 (10–15)	
6–10	15 (13–19)		16 (13–19)		12 (10–14)	
11–20	15 (14–17)		14 (10–17)		10 (8–13)	
>20	16 (14–19)		15.5 (13–17)		13 (10–15)	
Pediatric experience		0.737		0.416		0.325
1–5	16 (14–18)		16 (14–18)		13 (11–15)	
6–10	15 (13–18)		15 (13–17)		11 (8–14)	
11–20	16 (14–18)		16 (14–19)		12 (10–14)	
>20	16 (13–20)		16 (12–17)		12 (10–14)	
Nursing choice (rank)		0.174		0.588		0.084
1st	16 (14–20)		16 (13–18)		12 (9–15)	
2nd	14 (13.5–17)		15 (13.5–18.5)		11 (9–13)	
3rd	15 (13–18)		15 (13–17)		11 (10–14)	

**Table 5:** Associations of nurses’ characteristics with Self-Compassion subscales (Isolation, Mindfulness, and Over-Identification).

	Isolation		Mindfulness		Over-Identification	
	Median (IQR)	*p*-Value	Median (IQR)	*p*-Value	Median (IQR)	*p*-Value
Gender		0.829		0.155		0.482
Male	14 (12–16)		13 (10–15)		12 (10–14)	
Female	14 (12–16)		14 (12–16)		12 (11–14)	
Age (years)		0.005		0.090		0.005
≤35	15 (13–16)		13 (11–15)		11 (10–13)	
36–40	13 (11–15)		14 (12–15)		12 (11–14)	
41–50	14 (11–16)		14 (13–15)		12 (11–15)	
>50	14 (12–17)		15 (12–16)		14 (11–15)	
Marital status		0.904		0.542		0.045
Married/Cohabiting	14 (12–16)		14 (12–16)		13 (11–14)	
Single	14 (12–16)		13 (11–15)		11 (10–13)	
Divorced	13 (11–16)		13 (12–15)		13 (12–14)	
Number of children		0.508		0.147		0.041
0	14 (12–16)		13 (11–15)		12 (10–13)	
1	14 (12–16)		14 (12–16)		13 (11–14)	
≥2	14 (12–17)		14 (12–16)		13 (11–15)	
Total experience (years)		0.032		0.766		0.719
1–5	14 (13–16)		14 (12–17)		12 (10–15)	
6–10	14 (12–16)		13.5 (11–15)		12 (10–14)	
11–20	12 (11–15)		14 (13–15)		12 (11–14)	
>20	14 (12–16)		14 (12–15)		13 (11–14)	
Pediatric experience (years)		0.207		0.389		0.004
1–5	15 (13–17)		13 (12–15)		12 (9–13)	
6–10	15 (12–16)		13 (11–16)		11 (9–14)	
11–20	14 (11–15)		14 (12–16)		12 (11–14)	
>20	15 (13–17)		14 (12–16)		14 (11–15)	
Nursing choice (rank)		0.004		0.841		0.049
1st	15 (12–16)		13 (12–16)		12 (11–13)	
2nd	13.5 (11–16)		14 (11–15)		11 (9.5–13)	
3rd	16 (12–17)		14 (12–16)		13 (11–14)	

**Table 6:** Associations between self-compassion and nurses’ characteristics identified using multiple linear regression analysis (n = 208).

Total Self-Compassion β Coef (95% CI) *		*p*-Value
Number of Children		
0	Ref. Cat	
1	4.01 (0.02–7.99)	0.049
≥2	5.24 (0.93–9.54)	0.017
Self-Kindness β coef (95% CI)		
Age		
≤35	Ref. Cat	
36–40	0.02 (−1.37–1.41)	0.976
41–50	0.20 (−1.01–1.41)	0.742
>50	1.87 (0.50–3.25)	0.008
Common Humanity β coef (95% CI)		
Number of Children		
0	Ref. Cat	
1	0.58 (−0.30–1.46)	0.194
≥2	0.95 (0.00–1.90)	0.050
Isolation β coef (95% CI)		
Age (years)		
≤35	Ref. Cat	
36–40	−1.96 (−3.32–−0.61)	0.005
41–50	−1.05 (−2.54–0.44)	0.167
>50	−0.72 (−2.54–1.10)	0.436
Total nursing experience		
1–5	Ref. Cat	
6–10	−0.50 (−1.90–0.90)	0.482
11–20	−0.34 (−1.92–1.24)	0.673
>20	0.10 (−1.79–1.99)	0.918
Nursing choice (rank)		
1st	Ref. Cat	
2nd	−0.78 (−1.80–0.25)	0.135
3rd	−2.29 (−3.67–−0.91)	0.001
Over-Identification β coef (95% CI)		
Age (years)		
≤35	Ref. Cat	
36–40	−0.46 (−1.96–1.04)	0.546
41–50	−0.12 (−1.65–1.41)	0.874
>50	−0.07 (−1.97–1.82)	0.940
Marital status		
Married/Cohabiting	Ref. Cat	
Single	0.28 (−1.10–1.65)	0.690
Divorced	0.76 (−1.06–2.57)	0.411
Number of children		
0	Κατ.Aναφ.	
1	0.76 (−0.63–2.14)	0.283
≥2	1.36 (−0.14–2.86)	0.076
Pediatric experience (years)		
1–5	Ref. Cat	
6–10	−0.81 (−2.19–0.56)	0.244
11–20	−0.51 (−1.96–0.93)	0.482
>20	0.59 (−1.22–2.41)	0.519
Nursing choice (rank)		
1st	Ref. Cat	
2nd	−0.46 (−1.61–0.68)	0.425
3rd	−1.90 (−3.55–−0.26)	0.024

## Data Availability

The data sets generated during the current study are available from the corresponding author on reasonable request. The datasets generated and analyzed during the current study are not publicly available due to participant confidentiality. During the informed consent process, participants were assured that their data would remain strictly confidential and be accessed exclusively by the primary researcher.
